# The Possibilities of Antitoxin in Diphtheria

**Published:** 1898-04

**Authors:** George Suttie

**Affiliations:** Harper Hospital Polyclinic Staff; Lecturer On Organic Chemistry, Detroit College of Medicine


### THE POSSIBILITIES OF ANTITOXIN IN DIPHTHERIA.

By GEORGE SUTTIE, M. D., PH. C.,

Harper Hospital Polyclinic Staff; Lecturer On Organic Chemistry, Detroit College of Medicine.

There is no difference of opinion now among medical men regarding the efficiency of diphtheria antitoxin. Statistics have abundantly proved the decreased death-rate resulting from its use, although there may be found an occasional doubter who refuses to believe any kind of evidence regarding this new remedy.

I have no intention in the following to do more than ascertain from the statistics at my command what is the percentage of deaths that has been observed in diphtheria under the use of antitoxin—being prompted to do so by a statement made by one of the speakers at the Sanitary Convention held lately in the city of Detroit. It was then said that the use of antitoxin had established an expectancy of from 13–14 per cent. as a death-rate, instead of the old-time rate of 30 per cent. and over without its use.

It is hardly necessary to make the statement here, but it is unhesitatingly reiterated that antitoxin has decreased the mortality of the disease under consideration. But was the death-rate mentioned a fair average? Does it represent the best obtainable from antitoxin? The writer thinks not. The percentage given was ascertained to be the death-rate observed in the entire city of Detroit during the period of the first few months' trial and where the greater number of cases occurred among the careless, unhygienic poor, antitoxin being furnished in these cases gratuitously by the city.

To those familiar with the squalor of the poor in a large city it is not necessary to say that any medicament for any disease whatever does not receive its most favorable test under the conditions which obtain among this class of our population. It is certainly much in its favor that it was observed to be of service at all in the midst of so generally forbidding environment. Antitoxin must be used early in the diphtheritic attack, at least before the fourth day, to get the best results—so accurate hospital observations have taught us—and this fact physicians themselves did not realize at first.

The class of people, however, of whom we speak are proverbially the slowest to call a physician, even if it be the city physician, whose service is without cost to them, and it frequently happens that their call for help is too late for antitoxin to save the cardiac centers from involvement in the general toxemia. What percentage of deaths, then, may be expected from the use of antitoxin in diphtheria under hygienic conditions, and with the antitoxin administered so soon as diagnosis is made?

Having had something to do with the introduction of antitoxin for the use of the Contagious Department of Harper Hospital of this city, and having watched its administration from the beginning, the writer believes that a very much better percentage of result is attained with the early use of antitoxin in situations where hygienic conditions prevail, such as are found in a well-conducted hospital or even in the average private practice.

Antitoxin was first used in Harper Hospital in the fall and winter of 1894, in a sporadic, tentative, way. Forty-four cases of diphtheria were treated with the Aronson and Behring's serum, with four deaths (exclusive of three moribund on admission) and a death-rate accordingly of 9.1 per cent., when it happened that the supply of these became exhausted. At this juncture Parke, Davis & Co.'s diphtheria antitoxin was employed and found to be fully equal to any of the serums which had been previously employed; indeed, this is putting it too mildly, for not only did fewer disagreeable sequelæ follow its hypodermic administration, but cases progressed manifestly better under it. Thus in the remaining period of 1895 there were twenty-five cases, all of whom were treated with this serum (with exclusion of one moribund on admission). There was one death among these, the death-rate accordingly being 4.1 per cent.* Four were tracheotomy cases with four recoveries.

In 1896 there were 112 patients admitted to the diphtheria ward of the Contagious Hospital, twelve being subjected to intubation, six to tracheotomy, with only five deaths, "three of these being moribund cases when brought to the hospital, dying within a few hours of their arrival."† There was, therefore, a mortality of 1.8 per cent. These cases also received the Parke, Davis & Co.'s serum.

For the present year, 1897, up to December, there have been treated ninety cases of diphtheria in the hospital, with six deaths. One case was certainly moribund on admission, another may be called doubtfully so, but without excluding the latter, the death-rate was 5.6 per cent. The antitoxin used was the same.

The notable diminution in the death-rate as above given led the Detroit Board of Health to decide to furnish diphtheria antitoxin gratuitously to patients too poor to pay for it, this also including patients under charge of the city physicians, poor patients at Harper Hospital sent there by the Board of Health, at the Woman's Hospital and at the Protestant Orphan Asylum. This was done from May 1, 1896, and the number of patients so treated up to February 28, 1897, close of the official year,‡ was as follows:

*Vide *Therapeutic Gazette*, February 1896 detailing these cases.
†*Harper Hospital Bulletin*, February, 1897.
‡Report of Board of Health, Detroit Mich., for twelve months, ending February 28, 1897, p. 8.

| | Cases. | Deaths. | Mortality Rate. |
|---|---|---|---|
| With Antitoxin | 374 | 47 | 12.58 per cent. |
| Without Antitoxin | 467 | 163 | 34.90 per cent. |

In continuation of this series of observations are the following results from figures not yet made public, but kindly furnished me by the Board of Health. From March 1, 1897, up to December, the following cases came either under the notice or care of the Board:

| | Cases. | Deaths. | Mortality Rate. |
|---|---|---|---|
| With Antitoxin | 305 | 32 | 10 49 per cent. |
| Without Antitoxin | 632 | 192 | 30.39 per cent. |

The antitoxin employed by the Detroit Board of Health has been from the first that of Parke, Davis & Co.

These figures go to show that as experience in its use accumulates and as both medical men and the public at large see the advantage arising from the use of antitoxin early in the diphtheritic invasion, there is attained a continued and steady improvement in results.

With patients in comfortable circumstances, assured of careful nursing, conscientious isolation, and the administration of a reliable antitoxin, there is very much to encourage the profession to expect a very close approach to the Harper Hospital figures—that is to say, a percentage of death not to exceed six or seven. This would certainly lift diphtheria out of the bad repute it has had hitherto of being one of our most fatal diseases.—Louisville Medical Monthly.

